# Understanding the Complexity of Sleep Disturbances in ASD: From Mechanisms to Management

**DOI:** 10.3390/diagnostics16111727

**Published:** 2026-06-03

**Authors:** Adelina Glangher, Ina-Ofelia Focsa, Vanda Roxana Nimigean, Florentina Ionela Linca, Doina Ioana, Sorina Mihaela Papuc, Alina Erbescu-Dobre, Catrinel Iliescu, Carmen-Adella Sirbu, Magdalena Budisteanu

**Affiliations:** 1 Psychiatry Research Laboratory, Prof. Dr. Alex. Obregia Clinical Hospital of Psychiatry, 041914 Bucharest, Romania; adelina.huza@drd.umfcd.ro (A.G.); florentina-ionela.linca@fpse.unibuc.ro (F.I.L.); ioana.p.doina.m20@s.fpse.unibuc.ro (D.I.); laborator.cercetare.psihiatrie@spital-obregia.ro (M.B.); 2 Doctoral School, Faculty of Medicine, Carol Davila University of Medicine and Pharmacy, 030167 Bucharest, Romania; 3 Department of Medical Genetics, Faculty of Medicine, Carol Davila University of Medicine and Pharmacy, 020021 Bucharest, Romania; 4 Department of Oral Rehabilitation, Faculty of Dentistry, Carol Davila University of Medicine and Pharmacy, 050037 Bucharest, Romania; 5 Department of Special Psychopedagogy, Faculty of Psychology and Educational Sciences, 0506578 Bucharest, Romania; 6 Medical Genetics Laboratory, Victor Babes National Institute of Pathology, 050096 Bucharest, Romania; ela.papuc@ivb.ro (S.M.P.); alina.erbescu@ivb.ro (A.E.-D.); 7 Doctoral School, Faculty of Biology, University of Bucharest, 050095 Bucharest, Romania; 8 Clinical Neuroscience Department, Carol Davila University of Medicine and Pharmacy, 050474 Bucharest, Romania; catrinel.iliescu@umfcd.ro (C.I.);; 9 Pediatric Neurology Department, Prof. Dr. Alex. Obregia Clinical Hospital of Psychiatry, 041914 Bucharest, Romania; 10 Academy of Romanian Scientists, 050045 Bucharest, Romania; 11 Department of Genetics, Faculty of Medicine, Titu Maiorescu University, 031593 Bucharest, Romania

**Keywords:** autism spectrum disorder, sleep disturbances, circadian rhythm, insomnia, parasomnias, altered sleep architecture, sleep-related movement disorders, sleep-related breathing disorders, melatonin

## Abstract

Sleep disturbances represent one of the most frequent and clinically significant comorbidities in children with autism spectrum disorder (ASD), affecting approximately 50–80% of individuals. Clinically, these disturbances encompass a broad spectrum of disorders, including insomnia, parasomnias, sleep-related movement disorders, and sleep-related breathing disorders, commonly presenting with prolonged sleep latency, frequent nocturnal awakenings, reduced total sleep time, and alterations in sleep architecture. Circadian rhythm dysregulation, abnormalities in neurotransmitter systems such as GABA and serotonin, and altered melatonin signaling have been consistently implicated. These processes may reflect underlying genetic and metabolic influences affecting circadian clock regulation and synaptic function. The management of sleep disturbances in ASD requires a comprehensive approach combining behavioral strategies, caregiver education, and sleep hygiene interventions, while pharmacological options, particularly melatonin, may be considered when non-pharmacological measures are insufficient. Understanding the multifactorial mechanisms underlying sleep disturbances in ASD is essential for improving early recognition and developing individualized therapeutic strategies. This review synthesizes current evidence on the prevalence, biological mechanisms, clinical manifestations, and management of sleep disturbances in ASD, providing an integrated perspective for both clinicians and researchers.

## 1. Introduction

Sleep disturbances (SDs) are among the most prevalent and disabling comorbidities in children with autism spectrum disorder (ASD) [1,2,3]. These difficulties typically involve problems with the quality, duration, or timing of sleep and may have a substantial impact on daytime distress and impaired functioning [4,5].

Autism spectrum disorder (ASD) is a neurodevelopmental condition of childhood characterized by persistent difficulties in social communication and interaction, associated with restrictive and repetitive behaviors [6,7]. Over time, there has been growing interest among researchers in identifying associated conditions that may aggravate core symptoms and influence developmental outcomes [8]. Among these, one of the most well-studied causes of daily functional impairment is SDs, which affect learning abilities, emotional regulation and social engagement [9,10]. SDs have been suggested to correlate with the severity of ASD symptoms [11,12]. Once addressed, significant improvements in daily functioning and in quality of life may be observed. In clinical practice, sleep problems are often among the first concerns reported by caregivers and frequently represent a source of significant family stress [13,14].

SDs in ASD manifest as a wide spectrum of features, including difficulties in the quality, timing, and amount of sleep. According to the third edition of the International Classification of Sleep Disorders (ICSD-3), more than seventy sleep-related conditions are recognized, ranging from insomnia disorders and sleep-related breathing disorders to central hypersomnolence disorders, circadian rhythm sleep–wake disorders, parasomnias, and sleep-related movement disorders [15].

The mechanisms underlying sleep disturbances in ASD are multifactorial and reflect the interaction of biological, circadian, genetic, sensory, behavioral, and environmental influences [16,17]. Difficulties in emotional regulation, heightened arousal, sensory sensitivities, and rigid bedtime routines interact with neurobiological vulnerabilities such as altered melatonin secretion, disrupted circadian signaling [18,19], neurotransmitter imbalance [7,20], genetic variants [21,22], and metabolic abnormalities [23,24].

Sleep disturbances in ASD are highly prevalent and present with marked clinical heterogeneity [16,17]. Their impact extends beyond the child, affecting family functioning and daily routines [12,13,25]. Thus, the medical community shows increasing interest in studying sleep disturbances in ASD patients, aiming for a better understanding of the underlying mechanisms and the development of optimized management strategies [19,26,27]. This review provides an integrative overview of current evidence on prevalence, biological mechanisms, clinical manifestations, and therapeutic approaches, highlighting the complexity of sleep disturbances in ASD, from mechanisms to management.

## 2. Material and Methods

This article summarizes the current scientific literature on sleep disturbances in autism spectrum disorder (ASD), focusing on prevalence, neurobiological mechanisms, psychiatric and developmental consequences, and management strategies. A literature search was conducted using PubMed, Scopus, and Web of Science for articles published in English between 2010 and 2025. The search included systematic reviews, meta-analyses, clinical studies, and key experimental studies relevant to sleep dysfunction in individuals with ASD. Search terms included combinations of “autism spectrum disorder,” “ASD,” “sleep disturbances,” “insomnia,” “circadian rhythm,” “melatonin,” “neurotransmitters,” “genetics,” “psychiatric comorbidities,” and “sleep management.” Additional relevant studies were identified through manual screening of reference lists. Studies were selected based on their relevance to sleep disturbances in ASD, with a focus on publications addressing prevalence, biological mechanisms, clinical manifestations, and management. Priority was given to recent studies, systematic reviews, and articles with clear methodological design. Articles were excluded if they were not relevant to the topic, lacked sufficient methodological clarity, or did not provide meaningful insight into the biological, clinical, or therapeutic aspects of sleep regulation. To reduce potential selection bias, studies were identified through multiple databases and manual screening of relevant references. Given the broad and evolving nature of this topic, the article was designed as a narrative review rather than a systematic review; therefore, no formal quality assessment or quantitative synthesis was performed.

## 3. Prevalence

Over the past decade, multiple studies have shown that SDs occur at highly elevated rates in children with ASD, with prevalence estimates ranging from 50% to 80%, depending on age, cognitive functioning, comorbid conditions, and the methods used for assessment [28,29,30]. In contrast, sleep problems affect approximately 20–30% of typically developing children, highlighting the considerable vulnerability of the ASD population. Insomnia symptoms, particularly prolonged sleep latency and frequent nocturnal awakenings, represent the most frequently reported concerns, affecting up to 70% of children with ASD [31,32].

Between 40% and 60% of children with ASD demonstrate measurable abnormalities on polysomnographic studies, such as reduced rapid eye movement (REM) sleep, increased N2 or slow-wave sleep, and greater REM fragmentation [33]. Parasomnias such as confusional arousals, sleepwalking, night terrors, and enuresis have been variably reported in ASD cases, at a notably higher rate than in the general pediatric population. In a large multicentric study, parasomnias were observed in up to 8% of individuals, whereas other studies documented parasomnias, particularly sleep terrors or confusional arousals, in up to 60% of children with ASD [34,35]. Enuresis has been reported in approximately 14% of adolescents and young people with ASD [36].

In polysomnographic studies, sleep-related movement disorders, including periodic limb movements and restless legs syndrome, have been identified as more frequent in children with ASD compared with typically developing children (TDC) [9,26]. Sleep-related breathing disorders, particularly obstructive sleep apnea, are also more common in individuals with ASD, occurring in about 14 to 40% of cases, as reported in large clinical cohorts [10,27].

This wide heterogeneity reflects the diverse sleep phenotypes observed in ASD, shaped by sensory sensitivities, behavioral rigidity, variations in cognitive ability, co-occurring psychiatric or medical conditions, and environmental influences [23,37]. The consistently elevated prevalence across studies underscores the need for routine screening and early intervention [38].

Sleep is essential for neuroplasticity, cognitive development, memory consolidation, emotional regulation, and behavioral control. Disrupted sleep during childhood increases the risk of emotional dysregulation, attentional difficulties, learning impairments, and psychiatric comorbidities. Understanding sleep physiology and typical developmental patterns is therefore fundamental for identifying deviations seen in ASD [39,40].

## 4. Sleep Physiology and Development

Sleep consists of recurring cycles alternating between non-rapid eye movement (NREM) and rapid eye movement (REM) stages. These stages, distinguished through polysomnography (PSG) and EEG characteristics, support essential neurodevelopmental processes [41].

Across a typical night, REM and NREM alternate in a cyclical sequence, and the relative proportion of each stage evolves with age. Overall sleep duration decreases progressively across the lifespan [42]. During early childhood, a developmental period marked by rapid growth and intense neurocognitive maturation, children spend nearly half of the 24 h cycle asleep [43,44].

Moreover, the distribution of sleep stages changes considerably across childhood: newborns spend nearly 50% of their total sleep time in REM sleep, while by the age of two this proportion typically declines to around 25%. Slow-wave sleep (SWS) predominates during early childhood and gradually decreases toward adolescence, while circadian regulation becomes more stable with age. This maturation is marked by increasing regularity in melatonin secretion, improved sleep–wake timing, and consolidation of endogenous rhythms [41,45].

Further, external factors such as light exposure, daily routines, sensory input, and parental involvement shape sleep–wake patterns throughout childhood. These influences are especially relevant in children with neurodevelopmental conditions, who may be more sensitive to environmental variability and experience greater difficulty achieving stable sleep rhythms.

Infants are born with an immature sleep–wake rhythms that are initially connected to the maternal clock. The circadian system continues to develop across the late prenatal and early postnatal periods [46,47]. Children whose mothers reported altered sleep rhythms, stress, anxiety, or depression during pregnancy show a higher incidence of attention deficits, impulsivity, and mood disorders [46,48].

Observations of polysomnographic markers and stage-specific EEG features across developmental periods provide convergent evidence that sleep is integral to brain maturation and information processing [49].

## 5. Mechanisms Underlying Sleep Disturbances in ASD

SDs in ASD arise from a multifactorial interplay of biological, circadian, genetic, metabolic, sensory, and behavioral mechanisms. These overlapping pathways are likely to contribute to the wide heterogeneity of sleep patterns observed in the ASD population [37,39,40]. However, much of the evidence supporting these mechanisms is derived from experimental, associative, or indirect clinical studies, and direct causal relationships remain incompletely understood.

### 5.1. Circadian Rhythms

Circadian rhythms are self-sustained biological oscillations that regulate a wide range of physiological processes, enabling the organism to anticipate and adapt to regular environmental changes, particularly the alternation of light and darkness [50]. Numerous behavioral and physiological functions follow a 24 h pattern, including hormone secretion, core body temperature, and the sleep–wake cycle, reflecting the fundamental role of circadian timing in maintaining internal homeostasis [51].

These rhythms are primarily orchestrated by the suprachiasmatic nucleus (SCN), a specialized structure within the anterior hypothalamus that synchronizes internal oscillators with external cues, primarily light. Photoreceptive retinal ganglion cells convey light information to the SCN, which in turn helps regulate melatonin production by the pineal gland, lowers core body temperature, and prepares the brain for sleep. When this system functions optimally, melatonin secretion typically rises in the evening, sleep propensity increases, and physiological processes align smoothly with the environmental light-dark cycle [46,52,53,54,55].

In ASD, however, several components of circadian regulation appear to function atypically. A growing body of evidence suggests that children with ASD often exhibit delayed circadian phase timing, most commonly expressed as a delayed dim-light melatonin onset (DLMO) [4,56]. Although DLMO can vary across typically developing children, studies such as those by Martínez-Cayuelas and colleagues show that children with ASD not only have delayed melatonin onset but also significantly reduced nocturnal melatonin levels, suggesting an overall blunting of circadian output [33]. These alterations have been associated with prolonged sleep latency, bedtime resistance, irregular sleep–wake patterns, and fragmented sleep [33,57].

At the molecular level, disruptions in core clock genes, including *PER1*, *PER2*, *CLOCK*, and *NPAS2*, have been documented in ASD. Genetic association and expression studies suggest that deleterious variations and altered regulation in these circadian-related genes may contribute to the sleep disturbances frequently observed in individuals with ASD [58,59,60]. These genes are involved in coordinating the transcription-translation feedback loops that generate circadian rhythmicity in almost all cells of the body. Internal rhythms may become unstable or poorly synchronized across physiological systems when these loops are disrupted, contributing to inconsistent sleep timing and difficulties with sleep initiation or maintenance [61,62,63].

Circadian disturbances in ASD may be further amplified by environmental and behavioral factors. Children with ASD often exhibit heightened sensory sensitivities, rigid routines, or irregular evening activity, all of which can interfere with normal entrainment [12]. Limited morning light exposure, increased screen use before bedtime, and variability in daily structure may further desynchronize internal rhythms from external cues [23,64]. Specifically, exposure to blue light emitted by screens can inhibit melatonin release and delay circadian phase, thus contributing to delayed sleep onset [65].

**Figure 1:**
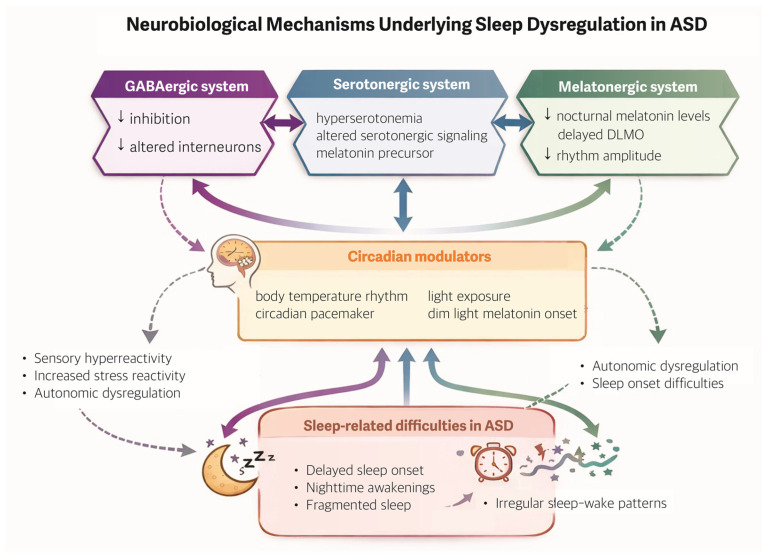
Neurobiological mechanisms underlying sleep disturbances in autism spectrum disorder. The figure illustrates the interaction between key neurochemical systems, including GABAergic, serotonergic, and melatonergic pathways, and circadian modulators such as body temperature rhythms, light exposure, and dim light melatonin onset (DLMO). Alterations in these systems, such as reduced inhibitory signaling, hyperserotonemia, and decreased nocturnal melatonin levels, interact with environmental and physiological factors, contributing to sensory hyperreactivity, autonomic dysregulation, and impaired sleep–wake regulation. These processes collectively lead to sleep-related difficulties in ASD, including delayed sleep onset, nighttime awakenings, fragmented sleep, and irregular sleep–wake patterns. Solid arrows represent proposed direct relationships, whereas dashed arrows represent indirect or contributory influences.

These findings suggest that circadian dysregulation in ASD results from a complex interplay between neurodevelopmental susceptibilities, altered melatonin physiology, genetic factors affecting the molecular clock, and external influences that disrupt the alignment between internal rhythms and the environment, as illustrated in Figure 1 [16,23]. This multifactorial disruption may underlie many of the sleep initiation and maintenance difficulties commonly observed in children with ASD [19,23].

### 5.2. Neurotransmitter and Neurochemical Imbalance

Sleep–wake regulation depends on the coordinated activity of several neurotransmitter systems, including GABAergic, serotonergic, cholinergic, and orexinergic pathways, many of which exhibit atypical functioning in children with ASD [66]. These neurochemical differences may contribute directly to the difficulties in sleep initiation, sleep maintenance, and overall sleep stability that are frequently observed in this population [26,51].

Among the systems most consistently implicated is the GABAergic pathway, which provides the inhibitory tone thought to be necessary for reducing cortical arousal and facilitating the transition into sleep, via hypothalamic projections targeting wake-promoting nuclei in the brainstem [66]. GABA plays a central role in promoting NREM sleep, synchronizing neuronal firing, and maintaining stable sleep architecture [66]. In ASD, disruption of GABAergic interneurons has been reported, and converging evidence from postmortem studies, genetic analyses, and neuroimaging indicates alterations in GABA receptor density and in genes involved in the synthesis and transport of GABA [67,68,69]. Reduced inhibitory signaling may result in a state of heightened physiological arousal, making it more difficult for children with ASD to settle at bedtime or to sustain consolidated sleep across the night [70,71].

The serotonergic system is also closely involved in sleep regulation. Serotonin modulates wakefulness and REM sleep and serves as the biochemical precursor of melatonin synthesis in the pineal gland [72]. One of the most replicated biological findings in ASD is hyperserotonemia, the presence of elevated whole-blood serotonin levels. Hyperserotonemia is present in over 25% of children with ASD and may have complex functional consequences. Disruptions in serotonin availability or signaling can alter REM regulation, influence arousal thresholds, and impair the normal conversion of serotonin into melatonin. These abnormalities may contribute to the irregular sleep–wake transitions and fragmented sleep commonly reported in ASD [19,73].

The melatonergic system, which is directly linked to circadian regulation and sleep initiation, may represent an important contributor to SDs observed in ASD. Melatonin is a hormone synthesized by the pineal gland and secreted primarily during the dark phase, with its production inhibited by light exposure [74,75]. In the central nervous system, melatonin attenuates the wake-promoting signal of the circadian clock, thereby facilitating sleep onset.

Numerous studies suggest that children with ASD tend to exhibit lower nighttime melatonin levels, delayed secretion profiles, or reduced amplitude of melatonin rhythms, including reduced nocturnal excretion of the melatonin metabolite 6-sulfatoxymelatonin [19,76]. These alterations may arise from deficits in key enzymes involved in melatonin synthesis, such as AANAT and ASMT, or from abnormalities in melatonin receptor function [77,78]. Beyond its chronobiotic role, melatonin also acts as a potent neuromodulator with antioxidant and neuroprotective properties. Given the high metabolic demands of neuronal activity, neuronal injury can trigger cascades involving excitotoxicity, oxidative stress, and inflammation [79]. Melatonin is considered a potent endogenous antioxidant, contributing to calcium homeostasis, limiting reactive oxygen species production, and protecting against ischemic neuronal damage [80]. Disruptions in melatonin dynamics may therefore have broad consequences for both sleep regulation and neurodevelopment [79,80].

Empirical evidence supports these associations in ASD. Martinez-Cayuelas and colleagues [33], in a cross-sectional study of 41 children and adolescents with ASD and 24 typically developing controls matched for sex, body mass index, and pubertal stage, reported alterations in circadian and sleep parameters, including dim light melatonin onset (DLMO). Before puberty, individuals with ASD showed a later DLMO, whereas during adolescence they exhibited an earlier decline in melatonin levels. In both ASD and typically developing children, later DLMOs were associated with delayed sleep onset, while in ASD participants a later daytime midpoint of temperature correlated with later DLMO. Moreover, later melatonin peak times and DLMO were associated with reduced general motor activity and decreased stability of melatonin secretory rhythms. These findings highlight the close relationship between melatonin rhythmicity and behavioral sleep characteristics in ASD [33].

Complementary findings were reported by Ping-I Lin and colleagues [81], who analyzed a cohort of 969 individuals with ASD genotyped and assessed for sleep disturbances using the Children’s Sleep Habits Questionnaire (CSHQ), with urinary melatonin levels measured in a subset of 219 participants. Their results revealed a significant correlation between urinary melatonin levels and CSHQ scores, with higher melatonin levels observed in ASD individuals presenting sleep difficulties [81].

Importantly, neurochemical imbalance in ASD does not occur in isolation. GABAergic, serotonergic, and melatonergic abnormalities interact with one another and with circadian regulatory mechanisms, creating a neurobiological landscape characterized by dysregulated arousal and impaired sleep–wake transitions. These mechanisms likely represent a probable, multifactorial model of sleep dysregulation rather than a direct explanatory pathway specific to ASD [16,23]. Heightened sensory reactivity, increased stress responsiveness, and autonomic dysregulation, features frequently described in ASD, are likely to further amplify these neurochemical vulnerabilities, destabilizing both sleep initiation and sleep maintenance [16,26]. Overall, the convergence of altered inhibitory signaling, atypical serotonin metabolism, and impaired melatonin physiology may provide a useful framework for understanding some of the sleep initiation difficulties, nighttime awakenings, and irregular sleep–wake patterns reported in ASD [16,23].

### 5.3. Metabolic and Energy Dysregulation

Growing evidence suggests that abnormalities in cellular energy metabolism represent an additional mechanism involved in SDs in ASD [82,83]. Neurodevelopment is an energy-intensive process, and even subtle alterations in metabolic pathways may have downstream effects on neuronal excitability, circadian regulation, and sleep architecture [24,84].

One of the metabolic pathways increasingly implicated in ASD is the canonical Wnt/β-catenin signaling pathway, a key regulator of neurogenesis, synaptic development, and tissue homeostasis within the central nervous system [85,86]. Multiple experimental and preclinical studies suggest that this pathway is hyperactivated in ASD, leading to a metabolic shift toward aerobic glycolysis, even in the presence of adequate oxygen availability [85]. This metabolic profile has been described as a “Warburg-like” effect and is associated with increased glucose consumption and elevated lactate production. Given that neuronal firing, synaptic plasticity, and circadian rhythm regulation require substantial mitochondrial energy supply, metabolic reprogramming toward aerobic glycolysis may alter neuronal excitability and disrupt sleep–wake regulatory circuits.

Such alterations in energy metabolism may influence sleep regulation [87]. The Wnt/β-catenin pathway itself exhibits circadian rhythmicity, with activity fluctuating across the 24 h cycle. Disruption of this rhythmic regulation may lead to a mismatch between cellular energy demand and circadian timing, potentially destabilizing sleep–wake transitions and sleep stage organization. Elevated lactate levels, in particular, have been associated with increased cortical excitability and impaired sleep depth, which may contribute to sleep fragmentation and reduced REM sleep, features that have been described in children with ASD. These associations are better interpreted within a multifactorial and probabilistic framework of sleep regulation, rather than as a direct explanatory pathway specific to ASD. Direct clinical validation in the context of sleep disturbances in ASD remains limited [88,89].

Metabolic dysregulation in ASD may also interact with neurotransmitter systems involved in sleep [24,89]. Evidence from experimental and translational studies suggests that altered glucose utilization and mitochondrial efficiency may influence the synthesis and turnover of neurotransmitters such as GABA and glutamate, further contributing to excitatory-inhibitory imbalance [24,90]. In addition, increased oxidative stress and impaired mitochondrial function, both reported in subsets of individuals with ASD [91,92], may exacerbate neuronal vulnerability during sleep, a period normally associated with metabolic clearance and cellular repair [89].

The close relationship between circadian rhythms and metabolism further underscores the relevance of metabolic pathways to sleep disturbances. Circadian clocks are known to regulate key metabolic processes, including glucose metabolism, mitochondrial activity, and redox balance, while metabolic signals can, in turn, feedback to influence circadian timing. In ASD, disruption of this bidirectional relationship may contribute to persistent circadian misalignment and atypical sleep architecture.

Overall, metabolic and energy dysregulation may represent an additional layer of complexity in the pathophysiology of sleep disturbances in ASD [23,93]. These alterations do not occur in isolation but interact closely with circadian mechanisms and neurotransmitter systems and are often shaped by underlying genetic susceptibilities that influence metabolic regulation, circadian timing, and synaptic function. This interplay may provide a biological framework linking cellular energy pathways to the genetic architecture of sleep disturbances observed in ASD [23,94].

### 5.4. Genetic Contributions

Genetic susceptibility in ASD is increasingly recognized as converging on molecular pathways that regulate circadian rhythmicity, neurochemical signaling, and cellular metabolism, thereby linking core aspects of ASD pathophysiology with sleep–wake regulation [60,95]. ASD is characterized by a multifactorial genetic architecture involving complex gene–gene interactions and epigenetic regulatory mechanisms, which may influence the expression and function of genes critical for circadian timing and neurotransmission [18,95,96].

Several genes constituting the core molecular circadian clock have been implicated in ASD, including *PER1*, *PER2*, *CLOCK*, and *NPAS2* [18,62]. These genes operate within transcriptional–translational feedback loops that generate endogenous circadian oscillations at the cellular level and ensure the temporal coordination of physiological and behavioral processes. Genetic variants or altered expression of these clock genes may disrupt the stability, amplitude, or phase of circadian rhythms, leading to misalignment between internal biological timing and external environmental cues. Such circadian instability has been associated with delayed sleep onset, irregular sleep timing, and increased sleep fragmentation, supporting the notion that intrinsic circadian vulnerability contributes substantially to sleep disturbances in ASD [62,63].

Besides their direct effects on circadian timing, alterations in core clock genes may also exert downstream influences on neurochemical systems involved in arousal regulation and sleep initiation [18,62]. Altered clock gene function can influence the rhythmic expression of genes regulating neurotransmitter synthesis, release, and receptor sensitivity, thereby affecting GABAergic inhibitory tone and serotonergic signaling. Disruptions in these systems may further compromise the balance between sleep-promoting and wake-promoting processes, amplifying arousal dysregulation and impairing the normal transitions between wakefulness and sleep [97].

Genetic alterations affecting the melatonin pathway represent an additional and closely related mechanism contributing to sleep disturbances in ASD [77,98]. Polymorphisms and mutations in melatonin receptor genes (*MTNR1A* and *MTNR1B*) may reduce receptor sensitivity or signaling efficacy, thereby diminishing the ability of endogenous melatonin to synchronize circadian rhythms and facilitate sleep onset [99]. Moreover, variants in genes encoding key enzymes involved in melatonin synthesis, such as *AANAT* and *ASMT*, have been reported in individuals with ASD and may underlie the reduced nocturnal melatonin levels, delayed dim-light melatonin onset, and decreased amplitude of melatonin rhythms frequently observed in this population [77,98].

Beyond canonical circadian and melatonergic pathways, genes involved in synaptic structure and plasticity have emerged as important contributors to sleep disturbances in ASD. One of the most extensively studied examples is *SHANK3*, a gene essential for postsynaptic scaffolding and the integrity of excitatory synapses. Deletions or mutations in *SHANK3* have been associated with significant sleep abnormalities, including prolonged sleep latency, reduced total sleep time, and altered sleep architecture [100].

Experimental evidence further supports a direct mechanistic link between SHANK3-related synaptic dysfunction and circadian regulation. In animal models, deletion of exon 21 in the *SHANK3* gene has been specifically associated with delayed sleep onset [100,101]. Moreover, sleep deprivation was shown to amplify differences in prefrontal cortex gene expression between *SHANK3* mutant and wild-type mice, leading to the downregulation of several key circadian transcription factors, including *Per3*, *Bhlhe41*, *Hlf*, *Tef*, and *Nr1d1*. Together, these findings indicate that *SHANK3* deficiency not only disrupts synaptic organization and network-level excitability but also interferes with molecular circadian mechanisms, particularly under conditions of sleep pressure, thereby linking synaptic integrity, circadian misalignment, and sleep–wake dysregulation in ASD [101,102].

Additional evidence implicates genes involved in neurotransmitter transport and arousal circuitry in sleep disturbances associated with ASD. Variants in genes regulating inhibitory and monoaminergic neurotransmission, including *SLC6A1*, *SLC6A3*, *VGAT*, *UBE3A*, *ARHGEF10*, *AHI1,* and *KCNQ3*, as well as histamine receptor genes *HRH1* and *HRH3*, have been associated with dysregulation of neural circuits controlling wakefulness and sleep stability. These alterations may affect GABAergic inhibition, dopaminergic signaling, and hypothalamic arousal systems, potentially increasing neural excitability and destabilizing the balance between sleep-promoting and wake-promoting pathways. Such alterations are associated with prolonged sleep latency, nocturnal awakenings, and reduced sleep duration. Collectively, these findings suggest that genetic disruption of neurotransmitter systems involved in arousal regulation represents an additional mechanism linking molecular genetics with the sleep–wake abnormalities frequently observed in ASD [103].

In addition to single-gene variants, large-scale genomic studies have identified copy number variations (CNVs) encompassing genes related to circadian regulation and insomnia that are more prevalent in individuals with ASD compared with TDC. These CNVs frequently span multiple genes and biological pathways, highlighting the polygenic and pleiotropic nature of sleep disturbances in ASD and suggesting that cumulative genomic burden may be a critical determinant of sleep phenotypes [104].

Supporting this view, Tesfaye and colleagues [105] systematically investigated the contribution of CNVs affecting circadian and insomnia-related genes to ASD susceptibility and sleep impairments in a large cohort comprising 5860 individuals with ASD, 2092 unaffected siblings, and 7509 typically developing controls. The authors identified a total of 335 rare CNVs involving circadian genes and 616 CNVs affecting insomnia-related genes. Both deletions and duplications targeting circadian genes were significantly overrepresented in individuals with ASD compared to controls and unaffected siblings, whereas only deletions involving insomnia-risk genes were associated with ASD. Importantly, beyond these gene-specific effects, CNVs affecting circadian and insomnia-related pathways as a whole showed a stronger association with ASD than variants located in unrelated genomic regions, reinforcing the notion that disruptions of sleep- and circadian-related genetic networks contribute meaningfully to ASD risk and associated sleep disturbances [105].

Taken together, these findings highlight the multifactorial and interdependent nature of sleep disturbances in ASD, arising from the interaction between circadian dysregulation, altered melatonin signaling, synaptic dysfunction, metabolic pathways, and broader genetic vulnerabilities [18,105]. Rather than reflecting a single genetic cause, sleep disturbances appear to arise from the cumulative effects of multiple genetic vulnerabilities interacting with neurodevelopmental processes and environmental influences. This complexity has important clinical implications and emphasizes the need for individualized clinical assessment and targeted management strategies [18,106].

## 6. Clinical Manifestations of Sleep Disorders in ASD

**Figure 2:**
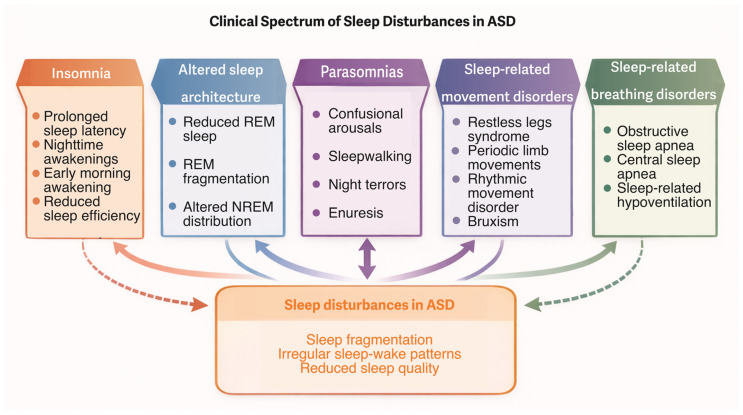
Clinical spectrum of sleep disturbances in autism spectrum disorder. The figure illustrates the main categories of sleep disorders observed in ASD, including insomnia, altered sleep architecture, parasomnias, sleep-related movement disorders, and sleep-related breathing disorders, along with their impact on sleep quality and daytime functioning, highlighting their potential overlap and cumulative impact on overall functioning. Solid arrows indicate direct manifestations or components of sleep disturbances in ASD, whereas dashed arrows indicate contributory or associated influences.

The complex mechanisms described above are reflected in the diverse clinical manifestations of SDs observed in children with ASD, which encompass a wide range of conditions, including insomnia, altered sleep architecture, parasomnias, sleep-related movement disorders, and sleep-related breathing disorders, all contributing to impaired sleep quality and daytime functioning as summarized in Figure 2.

### 6.1. Insomnia

Insomnia represents the most prevalent and clinically significant sleep disorder in children with ASD, with prevalence estimates ranging between 50% and 80%, substantially higher than those reported in typically developing peers [16]. Clinically, insomnia in ASD most commonly manifests as prolonged sleep latency, frequent nocturnal awakenings, early morning awakenings, reduced total sleep time, and decreased sleep efficiency. Consistent with these estimates, Galli and colleagues evaluated 100 children with ASD using standardized parental instruments, including the Early Life Sleep Behavior Questionnaire, the BEARS scale, and the short form of the Parenting Stress Index. Approximately 57% of participants met criteria for insomnia symptoms [32].

From a clinical perspective, insomnia is typically defined by a sleep latency exceeding 30 min, more than 30 min of wakefulness after sleep onset, sleep efficiency below 85%, or a total sleep duration shorter than 6.5 h. Studies examining sleep in children with ASD generally report a reduction in total sleep time (TST) compared to typically developing children [107,108,109]. TST is calculated as the difference between time in bed and the sum of sleep latency and wakefulness after sleep onset. Although total sleep time physiologically decreases across development, from approximately 16–20 h in the neonatal period to 14 h at one year of age, 12 h in preschoolers, 11 h during school age, and 9–10 h in adolescence, children with ASD appear to exhibit an earlier and more pronounced reduction. Specifically, ASD children begin to show decreased TST relative to typically developing controls by approximately 30 months of age, with a median reduction of 43 min reported by 81 months of age [110]. While the underlying causes of this reduction remain incompletely understood, factors such as poor sleep hygiene, increased access to electronic devices, anxiety or depressive symptoms, and reduced physical activity have been associated with shorter TST in this population.

Difficulties initiating sleep are particularly prominent in ASD. Under typical circumstances, sleep latency should not exceed 30 min [15]; however, many children with ASD require considerably longer periods to fall asleep despite appropriate bedtimes, often exceeding 30 min and in some cases extending beyond one hour [2]. These difficulties are frequently exacerbated by heightened physiological arousal, sensory hypersensitivity, anxiety, difficulty disengaging from repetitive behaviors or preferred activities, and behavioral rigidity surrounding bedtime routines, all of which may increase resistance to sleep initiation [12].

Sleep maintenance insomnia is also frequently reported and is characterized by multiple nocturnal awakenings and difficulty returning to sleep independently. Although some degree of wakefulness after sleep onset (WASO) is considered physiological, durations exceeding 30 min are generally considered pathological [15]. In children with ASD, nocturnal awakenings may be triggered by environmental sensitivity, parasomnias, or underlying circadian misalignment. Fragmented sleep may contribute to reduced sleep efficiency, defined as the ratio between total sleep time and time spent in bed, expressed as a percentage. For children, sleep efficiency values below approximately 74% are generally considered suboptimal [111,112,113,114].

Importantly, insomnia-related parameters, including prolonged sleep latency, increased WASO, and reduced sleep efficiency, have been consistently associated with greater behavioral difficulties in children with ASD [112,115]. Poor sleep has been linked to increased repetitive behaviors, reduced social engagement, heightened anxiety, and greater emotional lability. Moreover, chronic insomnia in ASD has been associated with impairments in attention, executive functioning, and learning, underscoring its clinical relevance beyond nighttime symptoms alone [12,21].

Notably, insomnia in ASD often appears to follow a persistent course without appropriate intervention. Unlike transient sleep difficulties commonly observed in early childhood, insomnia symptoms in children with ASD tend to be chronic and may be resistant to spontaneous resolution, emphasizing the importance of early identification and targeted intervention [27].

### 6.2. Altered Sleep Architecture

Sleep architecture undergoes marked developmental changes that closely parallel brain maturation. In neonates, REM sleep accounts for approximately 50–60% of total sleep time (TST), with even wider variability observed in premature infants, and REM sleep may occur at sleep onset [21,114]. By one year of age, the proportion of REM sleep is reduced by roughly half, while the amount of deep NREM sleep (N3) progressively increases throughout childhood, peaking during adolescence before declining to adult levels [111,116,117]. This developmental trajectory reflects the maturation of neural circuits involved in synaptic plasticity, emotional regulation, and cognitive processing [116].

Beyond subjective sleep complaints, objective alterations in sleep architecture have been consistently documented in children with ASD using polysomnography and actigraphy [16]. These alterations are thought to reflect underlying neurobiological and circadian dysregulation and may contribute to the non-restorative quality of sleep frequently reported in this population.

One of the most robust and reproducible findings in ASD appears to be a reduction in REM sleep. Compared with typically developing children, children with ASD tend to spend less time in REM sleep and proportionally more time in light and slow-wave sleep [118,119]. REM sleep in ASD is often characterized by increased fragmentation, reduced REM density, and frequent transitions between sleep stages [16]. Given the critical role of REM sleep in memory consolidation, emotional processing, and synaptic plasticity, these abnormalities may contribute to the cognitive, emotional, and behavioral difficulties observed during wakefulness [118].

Alterations in NREM sleep have also been described, including prolonged time spent in N2 sleep and increased slow-wave sleep (SWS), particularly in younger children with ASD. While SWS is generally considered restorative, an atypical distribution or excessive dominance of specific sleep stages may reflect delayed or altered patterns of brain maturation. Supporting this interpretation, EEG studies have reported differences in sleep spindle activity and slow-wave characteristics in ASD, suggesting atypical thalamocortical connectivity and altered synaptic organization [112,120].

Sleep stage instability, manifested as frequent arousals and micro-awakenings, represents another common feature of altered sleep architecture in ASD [118]. This fragmentation may reduce overall sleep efficiency and may exacerbate daytime sleepiness, irritability, and attentional difficulties. Notably, the extent of sleep architecture disruption has been reported to correlate with ASD symptom severity, cognitive functioning, and the presence of comorbid conditions such as anxiety or attention-deficit/hyperactivity disorder [16].

Circadian rhythm sleep–wake disorders (CRSWDs) have also been increasingly described in individuals with ASD and may contribute to irregular sleep–wake patterns. Delayed sleep–wake phase disorder appears to be particularly frequent and is characterized by a persistent delay in sleep onset and wake time relative to socially conventional schedules [121]. Objective investigations using actigraphy and circadian biomarkers have shown altered sleep–wake timing and reduced synchronization with environmental cues in children and adolescents with ASD. These disturbances are thought to reflect abnormalities in circadian regulation, including altered melatonin rhythms and impaired responsiveness to external environmental influences such as light exposure and daily routines. As a consequence, many individuals with ASD may exhibit delayed bedtimes, irregular sleep–wake cycles, and increased day-to-day variability in sleep timing, which may further exacerbate insomnia symptoms and daytime behavioral difficulties [122,123].

Overall, altered sleep architecture in ASD appears to reflect intrinsic neurodevelopmental differences rather than merely secondary consequences of behavioral sleep problems. Disruptions in the normal developmental organization of REM and NREM sleep likely compromise the restorative and regulatory functions of sleep, with downstream effects on cognition, emotion regulation, and daytime behavior [21].

### 6.3. Parasomnias

Parasomnias constitute another significant category of SDs seen in children with ASD, along with insomnia and changes in sleep patterns. These events occur either during sleep itself or when transitioning between sleep and wakefulness [26,124]. They are typically classified based on the sleep phase from which they emerge, either NREM- or REM-associated phenomena [15].

Children with ASD may experience parasomnias more frequently compared with the general pediatric population, which may reflect immature or dysregulated arousal systems during sleep. Reports describe both NREM- and REM-associated parasomnias occurring with variable frequency and clinical presentation. These phenomena may manifest either as standalone sleep disorders or as secondary symptoms of other medical conditions, including epilepsy or, less commonly, cardiac disorders. Potentially triggering factors include high body temperature, infectious diseases, substance exposure, medications, or sleep deprivation [26,125].

NREM parasomnias appear to be especially prevalent among younger ASD-diagnosed children and encompass confusional arousals, somnambulism (sleepwalking), night terrors, hypnic jerks, hallucinations occurring at sleep onset or upon awakening, exploding head syndrome, and sleep paralysis [126]. These phenomena generally emerge from deep sleep stages and involve incomplete awakenings, during which the child may seem bewildered, spatially disoriented, or non-responsive while displaying elaborate or contextually inappropriate actions [127]. Episodes typically end either with the child returning to sleep or achieving complete wakefulness. Several factors have been associated with an increased risk of NREM parasomnias in the ASD pediatric population, including insufficient sleep, inconsistent sleep timing, psychological stress, anxiety disorders, and resistance to bedtime routines [127,128].

Somnambulism is one of the most commonly documented parasomnias in ASD. During these episodes, children may walk while asleep and sometimes perform atypical actions, such as handling objects or carrying out familiar tasks. Aggressive behavior is rarely observed and typically arises in response to physical restraint or forced awakening. Episodes generally resolve spontaneously, either through full awakening or a return to sleep. In neurotypical children, sleepwalking and night terrors affect roughly 1–3% of the population, while children with ASD may demonstrate a notably higher rate of disoriented awakenings and parasomnia-associated behaviors [31].

REM-associated parasomnias have also been described in individuals with ASD, although they appear less common in childhood. These events may involve dream-enacting behaviors, including vocalizations, limb movements, rising to a seated position, or more elaborate physical actions that mirror dream narratives. Additional sleep-related phenomena occasionally reported in children include sleep paralysis, hypnagogic hallucinations, and other REM-related experiences, which typically occur during transitions between sleep and wakefulness. However, true REM sleep behavior disorder is generally considered rare in the pediatric population [26].

Although parasomnias are typically well-tolerated and self-limited, they may substantially interfere with daily family life functioning and increase parental stress, especially when episodes are frequent or unpredictable. Thus, clinical assessment is critical to distinguish parasomnias from nighttime seizure activity, behavioral wake-ups, or other sleep pathologies. In most cases, parasomnia episodes cease naturally and do not represent intentional, purposeful, or conscious actions [26,31,127].

### 6.4. Sleep-Related Movement Disorders

Sleep-related movement disorders are more frequently reported in children with ASD than in TDC and may contribute to sleep fragmentation and non-restorative sleep. These disorders often coexist with insomnia and circadian dysregulation, further complicating the clinical picture [129,130].

Among the most commonly described conditions are restless legs syndrome (RLS) and periodic limb movement disorder (PLMD). Children with RLS may experience uncomfortable or unpleasant sensations in the lower limbs, typically worsening in the evening or at rest, leading to increased bedtime resistance and difficulty initiating sleep. Given that young children and those with communication difficulties may struggle to describe these sensations, RLS is likely underdiagnosed in the ASD population [131].

PLMD is characterized by repetitive, involuntary limb movements during sleep that can cause frequent arousals and sleep fragmentation. Polysomnographic studies suggest that periodic limb movements occur at higher rates in children with ASD, contributing to reduced sleep efficiency and increased daytime sleepiness, irritability, and attention deficit [132].

Other movement-related phenomena observed during sleep in ASD include rhythmic movement disorder, such as body rocking or head banging, and sleep-related bruxism. These behaviors are particularly common in children with neurodevelopmental disorders and may reflect self-soothing strategies, sensory processing differences, or immaturity of motor inhibition during sleep [133].

Iron deficiency has been associated with both RLS and PLMD. Low ferritin levels, particularly below 50 ng/mL, are frequently identified in children with ASD presenting with movement-related sleep disturbances. Given the role of iron in dopamine synthesis and motor control, routine assessment of iron status may be considered in this context [134].

Overall, sleep-related movement disorders represent an important and often underrecognized contributor to disrupted sleep in ASD, warranting careful clinical evaluation and targeted management [129,130,133].

### 6.5. Sleep-Related Breathing Disorders

Sleep-related breathing disorders constitute another clinically relevant category of SDs in children with ASD. Among these, obstructive sleep apnea syndrome (OSAS) is the most prevalent form. Although OASA affects approximately 2–5% of the general pediatric population, prevalence estimates in children with ASD range from 14% to over 50% of children with ASD, depending on the study population and diagnostic methods [109,135,136].

OSAS in children with ASD has been associated with hypotonia, apraxia, craniofacial features, adenotonsillar hypertrophy, and obesity and is characterized by snoring, gasping, labored breathing during sleep, restless sleep, and frequent nocturnal awakenings. Daytime manifestations may include excessive sleepiness, hyperactivity, irritability, impaired attention, and worsening of behavioral problems [137].

Central sleep apnea syndrome (CSAS), although less common, has also been reported in children with ASD, particularly in those with associated neurological or genetic conditions affecting brainstem respiratory control. Additionally, sleep-related hypoventilation disorders may occur in the context of neuromuscular weakness or metabolic abnormalities, further contributing to sleep disruption [25,31].

Diagnosing sleep-related breathing disorders in children with ASD presents unique challenges, often being underdiagnosed. Overnight polysomnography may be limited by heightened sensory sensitivities, anxiety disorders, and challenges associated with the tolerance of monitoring apparatus. Consequently, clinical suspicion based on history context and caregiver reports plays a crucial role in recognizing children who may benefit from further assessment.

If untreated, sleep-related breathing disorders may have significant consequences for quality of life through worsening cognitive deficits, behavioral dysregulation, and an increased risk of cardiovascular complications. Early recognition and appropriate intervention are therefore essential components of comprehensive sleep management in ASD [14,26].

Clinically, sleep problems in ASD rarely present as a single entity. Insomnia, parasomnias, movement-related symptoms, and breathing abnormalities often overlap, which may amplify daytime impairment and complicate treatment planning. This makes careful phenotyping and individualized management essential [17,31].

## 7. Psychiatric Comorbidity and Developmental Consequences

SDs in ASD children have been associated with psychiatric comorbidities and developmental outcomes. Rather than representing isolated nighttime symptoms, disrupted sleep manifests bidirectional effects on emotional regulation; cognitive performance, such as learning and memory consolidation; attention; daily adaptive functioning; and behavioral stability.

Synaptic plasticity, which is vital for learning and memory, may be impaired by disrupted sleep. This is particularly concerning during critical periods of brain development in children with ASD, potentially contributing to long-term cognitive deficits [4].

Disorders in sleep regulation can lead to defective rest, which has been associated with psychiatric manifestations, including anxiety and depressive symptoms, even during early developmental stages [43,138]. Abnormal sleep regulation is a well-documented feature across several neurodevelopmental conditions, including ASD, Down syndrome, and ADHD, underscoring the shared vulnerability of developing neural systems to sleep disruption [43,139,140].

Poor sleep quality may influence emotional and behavioral dysregulation as evidenced by increased irritability, mood instability, and anxiety in children with ASD. It has been shown to amplify emotional reactivity and reduce tolerance to environmental stressors. Children experiencing chronic sleep deprivation may demonstrate more frequent tantrums, heightened aggression, and increased repetitive behaviors [12,21]. Several studies suggest that the severity of insomnia correlates with the intensity of core ASD symptoms, particularly social withdrawal and stereotyped behaviors. Moreover, anxiety disorders, highly prevalent in ASD, further exacerbate sleep difficulties, creating a vicious cycle in which anxiety worsens sleep and sleep disruption intensifies anxiety symptoms [12,26,141].

Sleep also plays a fundamental role in attention regulation, working memory, and executive functioning. Disrupted sleep in ASD has been associated with increased inattention, impulsivity, and reduced cognitive flexibility, potentially overlapping with or aggravating ADHD symptoms. Fragmented sleep and altered REM patterns may impair memory consolidation and learning processes, negatively affecting academic performance and adaptive skill acquisition [21,141,142].

Emerging evidence suggests that early-life sleep problems in children may be associated with later psychiatric disorders during adolescence and potential neurodegenerative conditions in adulthood [143]. Although the underlying mechanisms are not yet fully understood, longitudinal population-based studies have associated chronic childhood nightmares with an increased risk that may represent early markers of future neurological vulnerability [144,145,146]. While these findings are not specific to ASD, they provide a broader context for understanding the potential long-term impact of sleep disturbances. Ongoing SDs during critical developmental periods may disrupt normal brain maturation, potentially reducing resilience to neuropathological damage. These findings suggest that SDs in ASD should be viewed not only as symptomatic concerns but also as potential modifiers of long-term neurodevelopmental trajectories, although further research is needed to clarify these relationships [144].

Family functioning may also be significantly affected by SDs in children with ASD. Frequent nighttime awakenings, prolonged bedtime routines, and early morning awakenings of the children contribute to parental sleep deprivation and increased caregiver stress. In turn, this elevated caregiver stress has been associated with worsening behavioral difficulties in children, highlighting the bidirectional nature of sleep-related difficulties [13,25].

Given the heterogeneity of these clinical presentations, a structured and individualized approach to management is essential.

## 8. Management of Sleep Disturbances in ASD

Understanding the underlying mechanisms of SDs in ASD is essential for guiding clinical decision-making and selecting appropriate, individualized interventions. Targeted sleep-focused intervention strategies should be considered as a routine component of therapeutic management in children with ASD. In practice, treatment is most effective when it combines caregiver-guided behavioral strategies with targeted approaches for circadian misalignment and comorbid sleep disorders [27,147,148].

**Figure 3:**
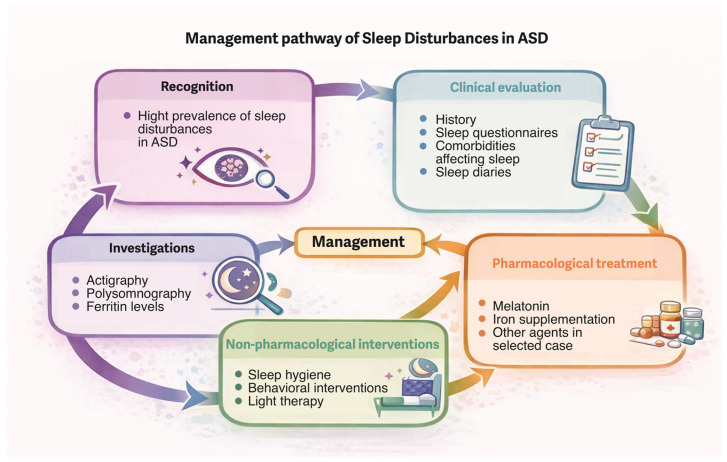
Management pathway for sleep disturbances in autism spectrum disorder. The figure illustrates a stepwise clinical approach, starting with recognition of the high prevalence of sleep disturbances in ASD, followed by clinical evaluation, including sleep history, standardized questionnaires, comorbidities, and sleep diaries. Further investigations such as actigraphy, polysomnography, and ferritin level assessment may be performed when indicated. Management includes non-pharmacological interventions, such as sleep hygiene, behavioral strategies, and light therapy, as first-line approaches, while pharmacological treatment, including melatonin and iron supplementation, may be considered in selected cases. The pathway highlights the importance of individualized and multidisciplinary management based on the underlying mechanisms and clinical presentation.

The management pathway is summarized in Figure 3, providing a structured and clinically applicable approach to the evaluation and treatment of SDs in ASD.

### 8.1. Non-Pharmacological Interventions

The presence of insomnia and circadian rhythm disorders should first be approached with non-pharmacological interventions.

Non-pharmacological strategies are generally recommended as the first-line approach for addressing insomnia and circadian rhythm disturbances in children and adolescents with ASD. Key components include psychoeducation and caregiver training, aimed at improving understanding of sleep needs, consistent routines, and behavioral factors influencing sleep [1,149]. Sleep hygiene practices include maintaining a comfortable dark and quiet sleep environment, minimizing evening exposure to blue light, encouraging independent sleep initiation, avoiding daytime naps, and limiting stimulating activities before bedtime. Behavioral sleep interventions include structured sleep routines, scheduled awakenings, gradually delaying bedtime until sleep onset occurs within 15 min and then progressively advancing bedtime. Sleep restriction techniques, involving a reduction in total time spent in bed to improve sleep consolidation, may also be considered in selected cases [150].

Lifestyle recommendations that may support improved sleep quality include restricting bed use exclusively for sleep; limiting screen time, particularly in the hours before bedtime; establishing relaxing pre-sleep routines; and avoiding stimulants, daytime naps, and high-intensity evening physical activity. These measures may help regulate sleep–wake patterns and enhance nighttime sleep continuity [151].

Light-based interventions, including exposure to natural or structured bright-light therapy, particularly exposure to morning light, may be beneficial for synchronizing the biological clock and stabilizing sleep–wake timing [149,152,153].

### 8.2. Pharmacological Interventions

Pharmacotherapy is typically considered only after non-pharmacological approaches have been attempted. Management should begin with an evaluation of potential contributing factors, such as anxiety, depression, narcolepsy, obstructive sleep apnea, periodic limb movements, or pain.

Melatonin remains the most commonly used pharmacological agent worldwide [28,152,154]. Immediate-release melatonin, 1–4 mg usually administered 20 to 60 min before bedtime, effectively reduces sleep onset latency. Prolonged-release formulations appear to have greater impact on total sleep duration and nocturnal continuity when administered 3–4 h before sleep as a chronobiotic [155,156,157,158].

Melatonin has demonstrated a favorable safety profile, with studies showing no significant adverse effects on growth, pubertal development, or withdrawal symptoms during use up to 10 weeks [28,159].

Assessment of iron status is also recommended, as low ferritin levels below 50 ng/mL may contribute to RLS and PLMS and may be more prevalent in individuals with ASD. Iron supplementation should be initiated when indicated [28,160,161,162].

Additional pharmacological options, such as gabapentin, certain antipsychotics, antidepressants, anticonvulsants, antihistamines, doxepin, or clonazepam, may be used in selected cases, though supporting evidence remains limited and should be considered carefully [28,163,164]. Targeted management is essential when specific sleep disorders are identified in children with ASD, as therapeutic approaches vary considerably depending on the underlying condition. In cases of restless legs syndrome or periodic limb movement disorder, evaluation of iron status is recommended, and iron supplementation should be initiated when ferritin levels are low, given the role of iron in dopaminergic function and motor regulation [134,160]. For children diagnosed with obstructive sleep apnea syndrome, treatment options may include adenotonsillectomy, particularly in the presence of adenotonsillar hypertrophy, or continuous positive airway pressure therapy in more persistent or complex cases [165,166,167]. Parasomnias, in contrast, often require behavioral reassurance, optimization of sleep hygiene, and stabilization of sleep schedules rather than pharmacological intervention [168,169]. Accurate diagnosis is therefore critical, as effective management depends on distinguishing among diverse sleep disorders that may present with overlapping symptoms but require fundamentally different therapeutic strategies [150].

Given the high prevalence and multidimensional impact of sleep disturbances in ASD, early identification of sleep problems may be an important component of clinical assessment. In practice, evaluating sleep alongside behavioral symptoms, psychiatric comorbidities, sensory sensitivities, and family context may help guide management decisions. Non-pharmacological approaches are commonly considered first-line, while pharmacological options, such as melatonin, may be used in selected cases. Such an approach has the potential to improve both sleep outcomes and overall daytime functioning in children with ASD.

## 9. Conclusions

Sleep disturbances represent one of the most prevalent and clinically significant comorbidities in children with ASD. As highlighted throughout this review, these disturbances arise from a complex interplay of circadian dysregulation, neurochemical imbalance, metabolic alterations, and genetic susceptibility. Rather than constituting isolated symptoms, sleep abnormalities in ASD reflect intrinsic neurobiological vulnerabilities that intersect with behavioral and environmental influences and manifest across multiple domains. Their consequences extend beyond sleep itself, influencing emotional regulation, cognitive development, psychiatric comorbidity, and overall quality of life for both children and their families.

Early identification and intervention are essential, as improving sleep may confer multiple benefits across different domains. Thus, effective management requires a personalized, multidisciplinary approach and careful consideration of underlying pathophysiological mechanisms.

## Data Availability

No new data were created or analyzed in this study. Data sharing is not applicable to this article.

## Abbreviations

The following abbreviations are used in this manuscript:

ADHD	Attention deficit hyperactivity disorder
AANAT	Aralkylamine N-acetyltransferase
ASD	Autism spectrum disorder
ASMT	Acetylserotonin O-methyltransferase
CNVs	Copy number variants
CRSWDs	Circadian rhythm sleep–wake disorders
CSAS	Central sleep apnea syndrome
CSHQ	Children’s Sleep Habits Questionnaire
DLMO	Dim light melatonin onset
DSM	Diagnostic and Statistical Manual of Mental Disorders
DSM-5	Diagnostic and Statistical Manual of Mental Disorders fifth edition
EEG	Electroencephalography
GABA	Gamma-aminobutyric acid
HPA	Hypothalamic–pituitary–adrenal axis
ICSD-3	International Classification of Sleep Disorders third edition
MNTR1A	Melatonin receptor 1A
MNTR1B	Melatonin receptor 1B
NREM	Non-rapid eye movement
OSA	Obstructive sleep apnea
OSAS	Obstructive sleep apnea syndrome
PLMD	Periodic limb movement disorder
PSG	Polysomnography
REM	Rapid eye movement
RLS	Restless legs syndrome
SCN	Suprachiasmatic nucleus
SDs	Sleep disturbances
SWS	Slow-wave sleep
TST	Total sleep time
WASO	Wake after sleep onset
